# Design of mid-infrared filter array based on plasmonic metal nanodiscs array and its application to on-chip spectrometer

**DOI:** 10.1038/s41598-021-91762-7

**Published:** 2021-06-09

**Authors:** Hwa-Seub Lee, Gyu-Weon Hwang, Tae-Yeon Seong, Jongkil Park, Jae Wook Kim, Won Mok Kim, Inho Kim, Kyeong-Seok Lee

**Affiliations:** 1 Center for Neuromorphic Engineering, Korea Institute of Science and Technology, Seoul, 02792 Korea; 2 Department of Materials Science and Engineering, Korea University, Seoul, 02841 Korea

**Keywords:** Nanophotonics and plasmonics, Mid-infrared photonics

## Abstract

Mid-infrared wavelengths are called the molecular fingerprint region because it contains the fundamental vibrational modes inherent to the substances of interest. Since the mid-infrared spectrum can provide non-destructive identification and quantitative analysis of unknown substances, miniaturized mid-infrared spectrometers for on-site diagnosis have attained great concern. Filter-array based on-chip spectrometer has been regarded as a promising alternative. In this study, we explore a way of applying a pillar-type plasmonic nanodiscs array, which is advantageous not only for excellent tunability of resonance wavelength but also for 2-dimensional integration through a single layer process, to the multispectral filter array for the on-chip spectrometer. We theoretically and experimentally investigated the optical properties of multi-periodic triangular lattices of metal nanodiscs array that act as stopband filters in the mid-infrared region. Soft-mold reverse nanoimprint lithography with a subsequent lift-off process was employed to fabricate the multispectral filter array and its filter function was successfully extracted using a Fourier transform infrared microscope. With the measured filter function, we tested the feasibility of target spectrum reconstruction using a Tikhonov regularization method for an ill-posed linear problem and evaluated its applicability to the infrared spectroscopic sensor that monitors an oil condition. These results not only verify that the multispectral filter array composed of stopband filters based on the metal nanodiscs array when combined with the spectrum reconstruction technique, has great potential for use to a miniaturized mid-infrared on-chip spectrometer, but also provide effective guidance for the filter design.

## Introduction

The mid-infrared (2–20 μm) is a spectral range in which most chemical molecules have fundamental vibrational modes and exhibit characteristic absorption bands depending on their molecular bonding state. Naturally, it is referred to as a molecular fingerprint region and technically very important because in this wavelength region unknown samples can be identified regardless of the states of matter i.e. gas, liquid, or solid, or qualitative and quantitative analysis is possible with high-selectivity for specific target molecules^1^. Plasmonic antennas and resonators tailored to a specific target wavelength have been often used to amplify spectroscopic molecular absorption signals through strongly enhanced light-matter interaction^2,3^.

Conventionally, benchtop equipment such as Fourier Transform Infrared (FTIR) spectrometer has been used for spectroscopic analysis of organic and inorganic materials^4^. In recent years, there is an increasing demand to develop a miniaturized IR spectrometer for on-site measurements in various applications such as environmental monitoring, industrial process line control, oil condition measurement, water and food quality inspection, and medical diagnosis^5–7^.

It is suggested that the most effective way to miniaturize the spectrometer is to replace the optical component responsible for light dispersion, such as conventional prism and diffraction grating, with a multispectral bandpass filter array and integrate it on a photodetector array^8^. Unlike FTIR and grating type spectrometers, these filter array-based spectrometers have the advantage of being robust and compact because they do not require moving parts and bulky dispersive optics.

Various optical filters with different physical principles have been proposed, and a typical one is a Fabry–Perot (FP) filter which uses the optical interference effect of a dielectric resonator placed between two reflective films^9^. It has the advantage that resonance wavelength is easily tuned by adjusting the resonator thickness. In addition, the bandwidth can be greatly narrowed by employing a distributed Bragg reflector. However, since all of these are achieved by modifying the vertical dimension, as the number of filters required to implement the spectrometer increases, the process steps for lithography are increased largely in proportion to the square of the number of dielectric resonant layers required. Therefore, the FP filters are not suitable for high-density integration and mass production.

A linear variable filter (LVF) is proposed to effectively solve this problem of process step increase and now commercially available^10–12^. The LVF is a wedge type FP filter in which the thickness of a dielectric resonant layer varies linearly in the lateral direction, thereby allowing the center wavelength of transmission bands to shift continuously along the wedge direction. However, linear nature restricts its use to one-dimensional (1D) integration. The process of consistently reproducing the slope that determines the spectral resolution of the filter is complex and unfavorable in terms of scalability. Interferometric filters such as FP filters inevitably generate multiple modes of resonance. This determines the free spectral range and may limit broadband operation. In addition, there is a limited amount of dielectric materials transparent in the mid-infrared which prevents the versatile design of filter stacks^13^.

Guided mode resonance (GMR) filters have been also proposed for on-chip spectrometer applications^14,15^. The GMR filters consist of a zero-order diffraction grating and an adjacent single-mode waveguide, with features of narrow bandwidth, high peak transmittance/reflectance, and good out-of-band rejection. It has the advantage of being able to control resonance wavelengths by changing the lateral geometrical parameters such as grating period and duty cycle as well as the vertical optical thicknesses of the waveguide and grating layers. However, the angular tolerance of the filter is quite poor, and there is a limitation in reducing the filter size due to the minimum number of grating periods required to ensure sufficient resonance.

Plasmonic metal nanohole array (MNHA) is a more typical example of nanophotonic filters that enable tuning of resonance wavelengths in broadband range through lateral dimension control without vertical structural modification^16–19^. The coupling between the surface plasmon wave (SPW) propagating on a thin metal surface and the grating mode from the nanohole array periodically perforated on the metal film results in the phenomenon of extraordinary optical transmission (EOT). Then the peak position is dominated by the period. It has a simple structure and material combination and is easy to integrate into a two-dimensional (2D) high-density array of multispectral filters. On the other hand, the broad nature of the EOT curves leads to considerable spectral overlap between neighboring filters, which makes it unsuitable for direct-readout operation of the spectrometer and requires digital signal processing (DSP) for spectrum reconstruction. Since the EOT occurs at the condition of momentum matching of propagating SPW and grating, MNHA filters are not defined with a single transmission band and accompany multiple modes which may cause a detrimental effect in determining the wavelength of light entering each cell of the photodetector array. In addition, the peak efficiency of transmission bands is also limited by the optical loss at the metal surface.

Pillar-type metal nanodisc arrays (MNDAs) with a periodic lattice geometry can be a good alternative for implementing filter-array based on-chip spectrometer. MNDAs exhibit a strongly enhanced reflectance peak due to the coupling between localized surface plasmons (LSPs) and the grating of metal nanodiscs^20–22^. This forms a deep dip in transmission and makes them act as a stopband filter. Its center wavelength is also strongly dependent on the lattice period. An infrared spectral reconstruction using a multiperiodic array of gold nanoblocks has been recently demonstrated^23^. In the present work, we also take notice of the filter characteristics of MNDAs with a periodic lattice geometry and present their potential for on-chip spectrometer applications. We employed the MNDA filters in the transmissive mode operation of an on-chip spectrometer. The effects of geometrical parameters and the optical properties of the constituent materials on the spectral response of the plasmonic nanodisc array filters were theoretically analyzed using the finite-difference time-domain (FDTD) simulation. The samples of multispectral filter array based on Al nanodisc arrays were fabricated using nanoimprint lithography and their transmission spectra in the mid-infrared region were measured by a Fourier transform infrared (FTIR) microscope. With the measured filter function, we tested the feasibility of target spectrum reconstruction through digital signal processing (DSP) based on a regularization method for an ill-posed linear problem and examined the effect of the number of filters on the spectral reconstruction resolution. As a more practical example, the application to the IR spectroscopic sensor that monitors the degree of oil aging was evaluated as well.

## Results and discussion

### Numerical simulation of the effect of geometrical parameters and metal selection on the transmittance spectrum of metal nanodisc array filters

**Figure 1:**
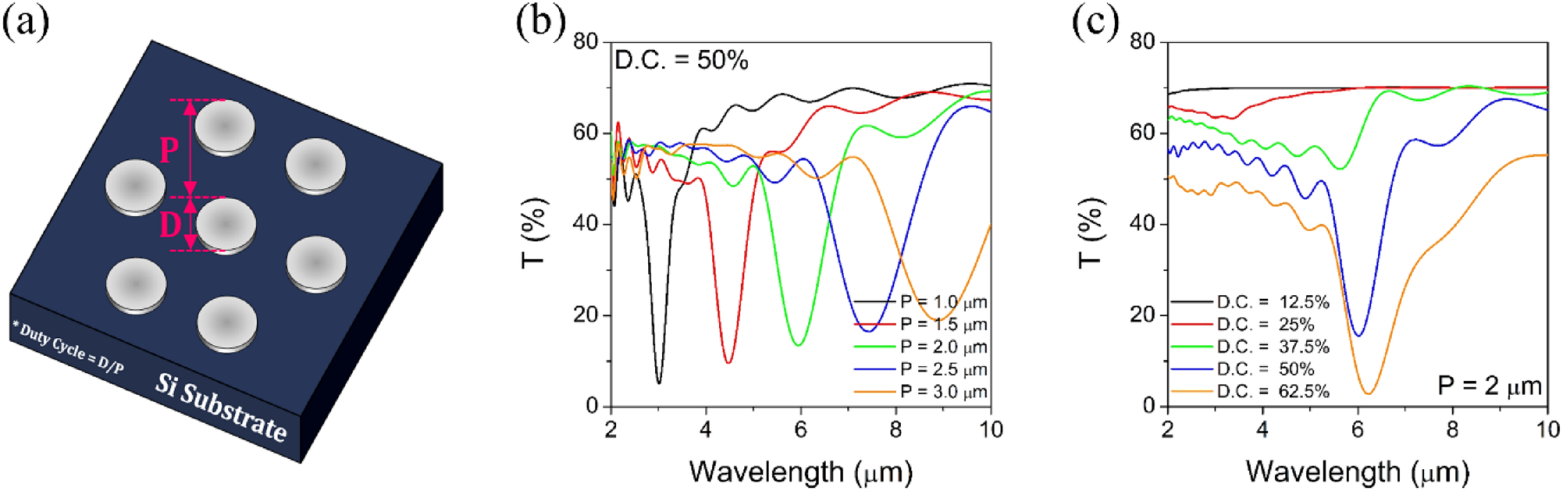
(**a**) Schematic diagram of a triangular lattice of metal nanodiscs and the simulated transmittance spectra as a function of (**b**) lattice period at a fixed duty cycle of 50% and (**c**) duty cycle at a fixed period of 2 μm. The thickness of Al nanodiscs is 50 nm.

The transmission spectra of metal nanodisc array filters were simulated using an FDTD method. Figure 1a shows a schematic geometry of the nanodiscs array with a triangular lattice used in the calculation. Here, Si which is transparent in the infrared wavelengths was used as a substrate, and the metal nanodiscs were assumed to be composed of Al. The effects of geometrical parameters such as lattice period (P), duty cycle (D.C. = D/P) defined as the ratio of disc diameter to period, and the height of nanodiscs on the optical transmission/reflection spectra were systematically analyzed.

Figure 1b,c show how the transmission spectra of the triangular lattice of the 50 nm thick Al nanodiscs change as a function of period and duty cycle, respectively. In Fig. 1b, the D.C. is fixed at 50%. Unlike the metal NHA structure, the MNDAs appear to have simple single stopband spectra having a relatively small linewidth and deep depth. This is because MNDAs exhibit a strongly enhanced reflectance peak due to the coupling between localized surface plasmons (LSPs) and the grating of metal nanodiscs, which forms a corresponding transmission dip^20–22^. Then, the resonance wavelength is predominantly dependent on the grating period. As shown in Fig. 1b, the center wavelength of the stopband is linearly red-shifted from 3.05 to 8.91 μm as P increases from 1 to 3.0 μm at the intervals of 0.5 μm.

Figure 1c shows the change in the transmission dip curve calculated with increasing D.C. from 0.125 to 0.625 at 0.125 intervals at the fixed grating period of 2 μm. When the D.C. is very small, it shows weak optical absorption due to the LSP resonance of isolated metallic nanodiscs. As the D.C. increases to more than 0.375, the central wavelength of the transmission dip is largely shifted to a long wavelength and the dip depth increases greatly. It is understood that this is because the dipolar coupling interaction between the LSPs and the periodic grating structure becomes stronger. At D.C. = 50%, the linewidth of the stopband is the narrowest. As the D.C. increases more than 50%, the depth of the transmission dip increases more and the center wavelength shifts to the longer wavelength, but the linewidth of the absorption band tends to be greatly broadened due to the near-field interaction between neighboring nanodiscs. Since the narrower and deeper stopband filters are advantageous for high spectral resolution, in this study, the D.C. is fixed at 50% afterward.

**Figure 2:**
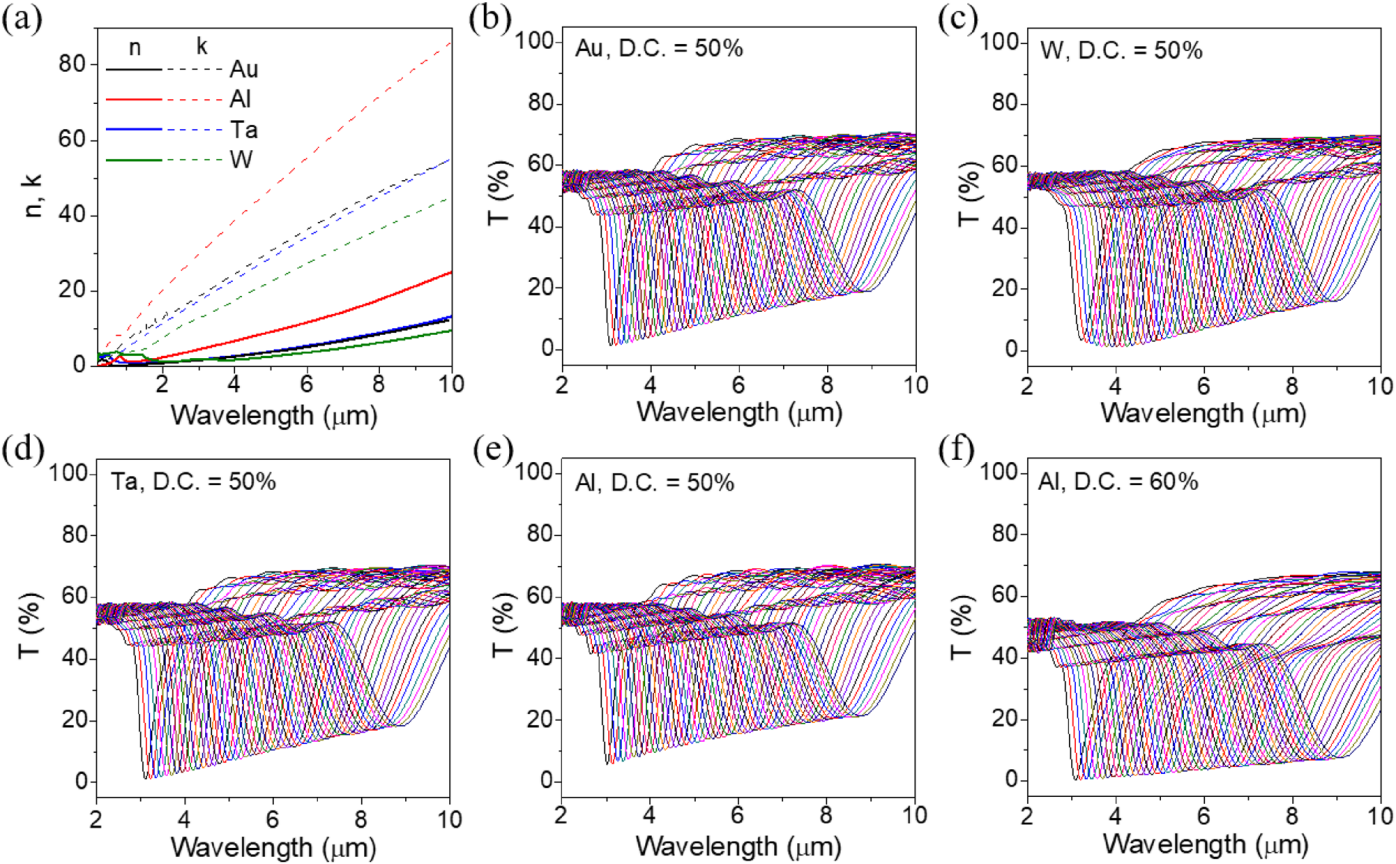
(**a**) Optical constants of various metals in the mid-infrared region and the simulated transmittance spectra for (**b**) Au, (**c**) W, (**d**) Ta, (**e**) Al nanodiscs array with a duty cycle of 50%, and (**f**) Al nanodiscs array with a duty cycle of 60%.

The selection of a metal constituting the metal nanodisc array may be an important factor. Unlike in the visible and near-infrared region where noble metals such as Al, Ag, Au, and Cu are preferred, refractory metals such as Ta, W, etc., whose optical behavior follows the Drude free electron model in the mid-IR region, can be also used as alternative plasmonic materials^24^. Figure 2a plots dispersion relations of optical constants for noble and refractory metals, which were taken from Palik’s handbook^25^. It is observed that the overall dispersion characteristic of refractory metals is very similar to that of the noble metals, which have a large extinction coefficient while the refractive index is kept low. This indicates that the refractory metals act as Drude metals in the mid-IR wavelength region. These metals not only have excellent thermal and mechanical stability but also have an advantage in that they do not require an additional adhesion layer owing to their strong inherent adhesion to the common substrates such as Si and SiO_2_.

The effect of the selection of metals on the IR transmission spectrum of MNDAs was simulated by employing Au, W, Ta, and Al metals and the results are shown in Fig. 2b–e, respectively. It is assumed that the filter array consists of a total of 49 filters whose period varies from 1 to 2.92 µm at intervals of 40 nm. Interestingly, regardless of the type of metal, the MNDA filters exhibit almost similar spectral features except for slight differences in the shape and depth of transmission dip curves. They all form single transmission dip curves in the mid-IR range of about 3 to 10 µm, with their resonance wavelengths continuously red-shift in proportion to the period. It is also observed that the transmission dip level goes up at the long-wavelength region. This is thought to be attributed to an increase in the imaginary part of dielectric constant 2*nk* representing the optical loss, which results in weakening the dipolar interaction in the periodic array of metal nanodiscs. On the other hand, as shown in Fig. 2f, increasing the disc diameter accompanying an increase in D.C. enlarges the dipole moment of the metallic nanodiscs, which contributes to lowering the level of transmission dip at the expense of linewidth broadening due to the enhanced near-field interaction between the neighboring discs.

### Fabrication of multispectral filter-array based on periodic lattice of Al nanodiscs and measurement of filter transmittance function in mid-infrared region

**Figure 3:**
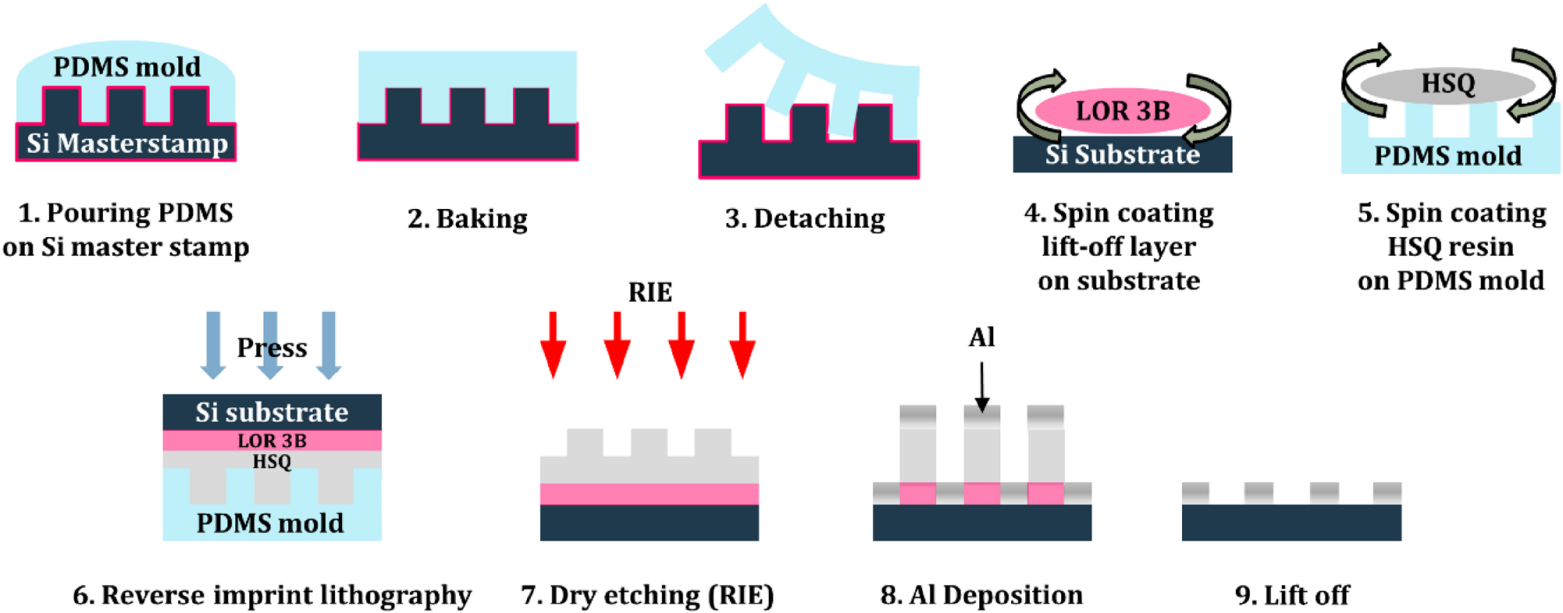
A schematic diagram of soft-mold reverse nanoimprint lithography (SRNIL) and subsequent lift-off process.

The multispectral filter array based on a periodic lattice of Al nanodiscs was fabricated by using soft-mold reverse-nanoimprint lithography (SRNIL) and a subsequent lift-off process. The filter arrays are designed and manufactured in 1D and 2D formats, respectively. The 1D filter array consisted of a total of 50 filters arranged linearly with a unit filter size of 200 µm × 2000 µm, and the 2D filter array consisted of a total of 49 filters with a unit filter size of 400 µm × 400 µm in a 7 × 7 array. The period of filters varies from 1 to 3 µm at 40 nm intervals and the duty cycle is fixed at 50%. Al, which is more easily deposited by e-beam evaporation than refractory metals, was selected as a metal for nanodisc array and double-side polished Si substrates were employed to ensure infrared transmission. The schematic procedure of fabricating the multispectral filter array of Al nanodiscs is illustrated in Fig. 3 and briefly explained here. The soft PDMS mold was fabricated by casting the PDMS precursor mixed in a ratio of 10:1 with a curing agent onto the Si master stamp, followed by a baking process. The cured PDMS mold was then carefully detached from the master stamp. The replica mold surface was spin-coated with an HSQ resin. Then the LOR 3B pre-coated Si substrate was placed face-down on it and pressed to transfer the patterned HSQ resin onto the substrate. After peeling off the mold, sequential dry etching steps were performed to remove the HSQ residue and the LOR 3B to open the substrate surface, on which a 50 nm thick Al layer was deposited by e-beam evaporation. Finally, the sample underwent a lift-off process to generate the Al nanodisc array. More details of the procedure are described in the experimental section.

**Figure 4:**
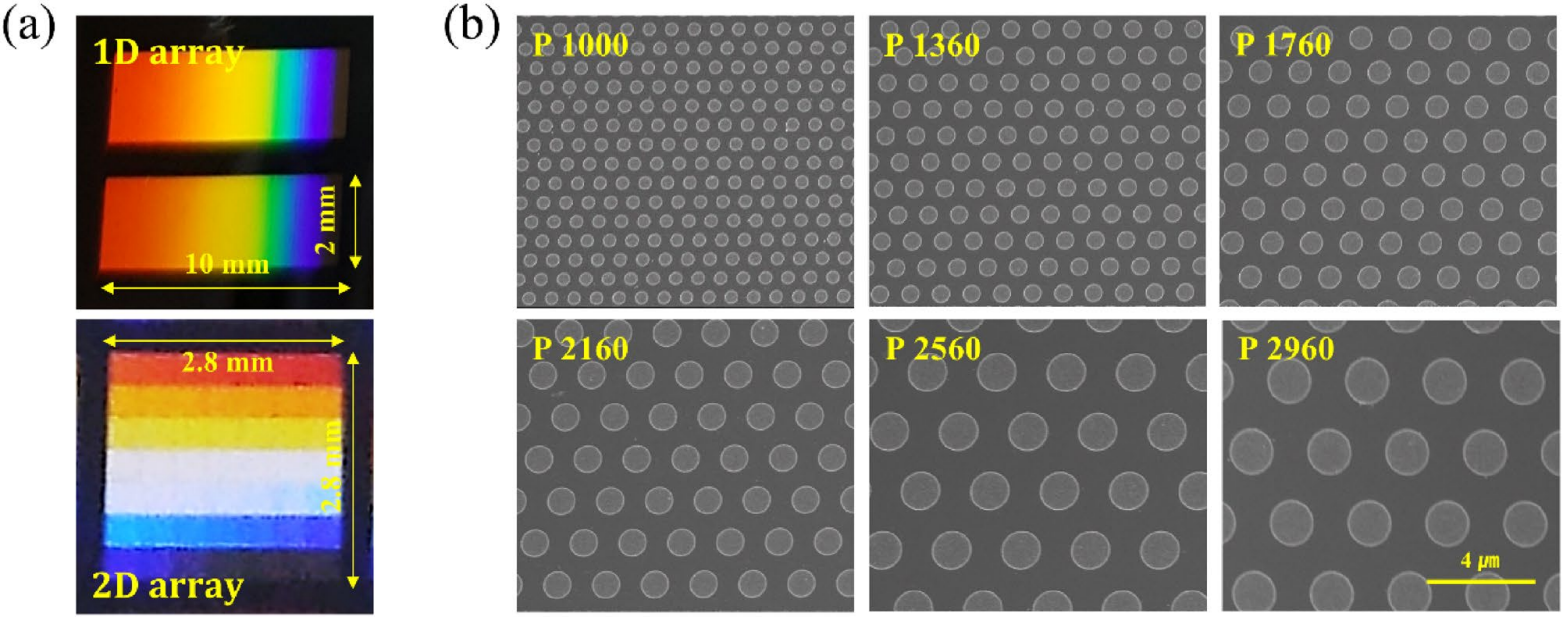
(**a**) The photographs of fabricated 1D (top) and 2D (bottom) filter array, and (**b**) plan-view SEM micrographs for several individual filters with different periods.

Figure 4 shows the photographs and scanning electron microscope (SEM) images of the multispectral filter array based on a periodic lattice of Al nanodiscs fabricated by SRNIL and the subsequent lift-off process shown in Fig. 3. The photographs for the 1D and 2D filter array samples (Fig. 4a) show gradually changing colors due to the grating effect which implies that the filter array composed of multi-periodic arrays of Al nanodiscs was successfully fabricated.

Figure 4b is the SEM images showing the surface microstructures of several individual filters with different periods constituting the multispectral filter array. This confirms that the triangular lattices of the Al nanodiscs array were successfully fabricated with varying periods. The measured periods were almost the same as that of a master stamp, but the duty cycle was observed to be increased by about 3.9% on average.

**Figure 5:**
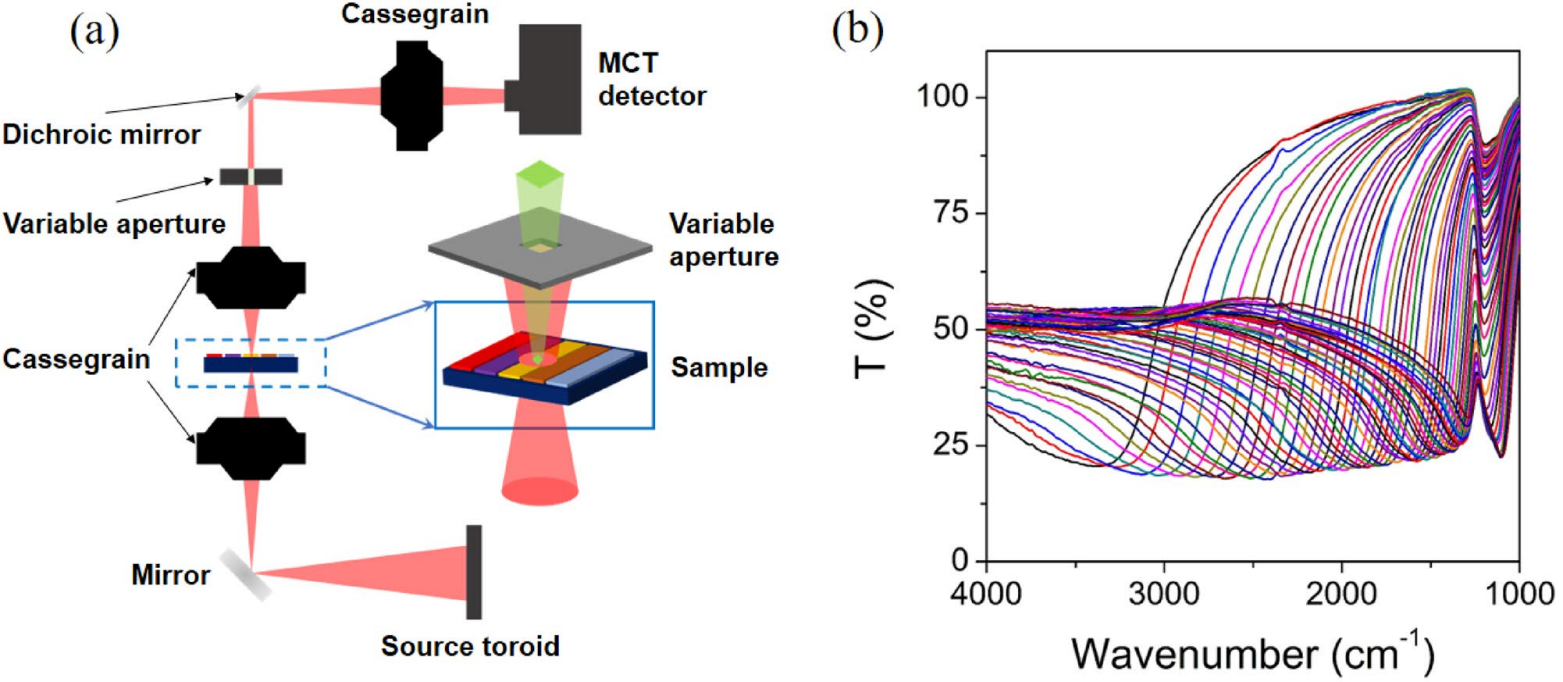
(**a**) A schematic diagram of FTIR microscope for the measurement of IR transmittance and (**b**) measured transmission spectra of Al nanodiscs array filters.

The transmission spectra of individual filters constituting the filter array were measured in the wavenumber range of 4000–1000 cm^−1^ (2.5–10 μm) using a Fourier Transform Infrared microscope. The 1D filter array shown in Fig. 4a was used for the measurement. The visible optical images of the 1D filter array were first monitored in the viewing mode of the FTIR microscope to position the sample to a measurement area of 200 μm × 200 μm. Then, the IR transmittance spectra of each of the total 50 filters were measured relative to a bare Si reference, as depicted in Fig. 5a. Figure 5b shows the measured transmittance spectra of the filter array. As the period of nanodiscs array increases, it is observed that the resonance dip positions of the transmission spectra were gradually shifted to the longer wavelength (lower wavenumber region). The baseline transmittance at the shorter wavelength side of the resonance dip curve shows a considerably lower value compared to the calculated one shown in Fig. 2. This is because for light with a wavelength smaller than the lattice period, higher-order diffraction components are created and these components do not reach the photodetector. The common transmission dips observed around 1100 cm^−1^ regardless of the period are ascribed to absorption originating from the Si substrate. Another major factor that causes the discrepancy from the theoretical curve can be found in the angle of incidence difference. Since the measurements were made using an FTIR microscope with a NA of 0.6, the incident angle is approximately 37° which may affect the spectral difference. In general, pillar type plasmonic nanodisc arrays are known to have superior angular tolerance compared with other metal nanohole arrays and guided-mode resonance filters that require momentum matching^26,27^. Nevertheless, in our simulation (now shown here), as the angle of incidence varies from 0° to 40°, the resonance wavelength redshifts just about 8% and the transmittance dip depth decreases by 17% for p-polarized light. The change is significantly reduced in the case of s-polarized light. Therefore, when applied to a real device, it is necessary to use the filter function measured under the operating conditions determined by the device configuration.

### Evaluation of plasmonic nanodisc array filter for on-chip spectrometer application using spectrum reconstruction method

**Figure 6:**
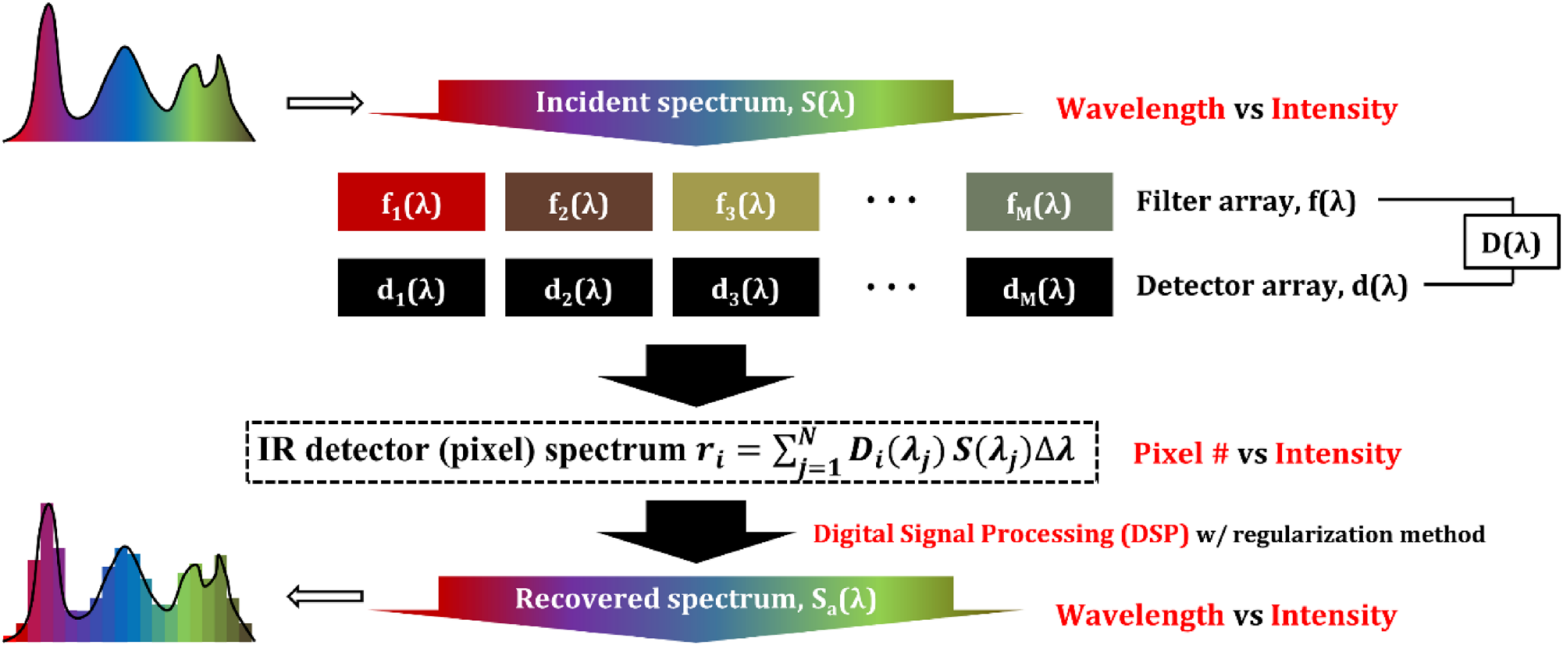
A schematic diagram illustrating the signal formation and spectral reconstruction process in the filter-array-based spectrometer.

When the target spectrum is measured through the array of multispectral filters integrated on an array of photodetectors, the output signals from the individual detectors are determined from the interaction between the source spectrum, the filter function, and the sensitivity function of the photodetector. The target spectrum is then reconstructed using a digital signal processing algorithm^28^. Figure 6 is a schematic diagram illustrating the signal formation and spectral reconstruction process in the filter-array-based spectrometer. Referring to Fig. 6, the detector signal r_i_ is expressed as Eq. (1) as a function of the filter transmittance f_i_(λ) for an i^th^ filter, the spectral responsivity of the detector d_i_(λ), and the target spectrum s(λ). Since the spectral responsivity of detectors are usually uniform over the array, f_i_(λ)d_i_(λ) is further simplified into D_i_(λ). This equation can be discretized into a linear matrix equation shown in Eq. (2). Here, n_i_ represents a system noise.

1 $$ r_{i} = \int\limits_{\lambda } {f_{i} \left( {\lambda_{j} } \right)} d_{i} \left( {\lambda_{j} } \right)s\left( {\lambda_{j} } \right)d\lambda_{j} = \int\limits_{\lambda } {D_{i} \left( {\lambda_{j} } \right)s\left( {\lambda_{j} } \right)} d\lambda_{j} $$

2 $$r = \left[ {\begin{array}{*{20}c} {r_{1} } \\ \vdots \\ \vdots \\ \vdots \\ {r_{M} } \\ \end{array} } \right] = \left[ {\begin{array}{*{20}c} {D_{1} \left( {\lambda_{1} } \right)} & \cdots & {D_{1} \left( {\lambda_{N} } \right)} \\ \vdots & \vdots & \vdots \\ {D_{i} \left( {\lambda_{1} } \right)} & \cdots & {D_{i} \left( {\lambda_{N} } \right)} \\ \vdots & \vdots & \vdots \\ {D_{M} \left( {\lambda_{1} } \right)} & \cdots & {D_{M} \left( {\lambda_{N} } \right)} \\ \end{array} } \right]\left[ {\begin{array}{*{20}c} {s\left( {\lambda_{1} } \right)} \\ \vdots \\ \vdots \\ \vdots \\ {s\left( {\lambda_{N} } \right)} \\ \end{array} } \right] + \left[ {\begin{array}{*{20}c} {n_{1} } \\ \vdots \\ \vdots \\ \vdots \\ {n_{M} } \\ \end{array} } \right] = DS$$

In general, the linear algebraic expression of Eq. (2) is defined to be an ill-posed problem because the number of filters (M) is smaller than the number of wavelength sampling (N), *i.e.,* it is in the underdetermined condition. Since there is no explicit inverse matrix of D(λ) with M ⨯ N (M < N) dimensions, the pseudo inverse may be used to recover the spectral signal but it is very vulnerable to small fluctuations or system noise, resulting in unstable results. In order to obtain a stable solution for this discrete ill-posed problem, regularization methods are commonly used.

The most representative method is Tikhonov regularization^29–33^. This method recovers the spectrum of the object to be analyzed by determining the solution s_*α*_, which minimizes the weighted sum of the residual norm and side constraint norm as shown in Eq. (3). Here, *α* is a regularization parameter which determines the weight of side constraint minimization with respect to the minimization of the residual norm, and there exists an optimal value for a robust solution. s* denotes an initial estimate of the solution. Singular value decomposition (SVD)^31,34^ and L-curve analysis method^29,35^ are used to adaptively determine the optimal regularization factor depending on the system and circumstances to be applied and hence enabling real-time spectrum reconstruction. Using these regularization methods, it is possible to recover the spectrum with a relatively high resolution while using a non-ideal filter array having a broad bandwidth. In addition to the general Tikhonov regularization, advanced sparse optimization algorithms^36,37^ have been attempted to obtain a more rigorous solution in the underdetermined cases very susceptible to noise.

3 $$ s_{a} = \arg \min \left\{ {\left\| {Ds - r} \right\|_{2}^{2} + \alpha^{2} \left\| {L\left( {s - s^{*}} \right)} \right\|_{2}^{2} } \right\} $$

**Figure 7:**
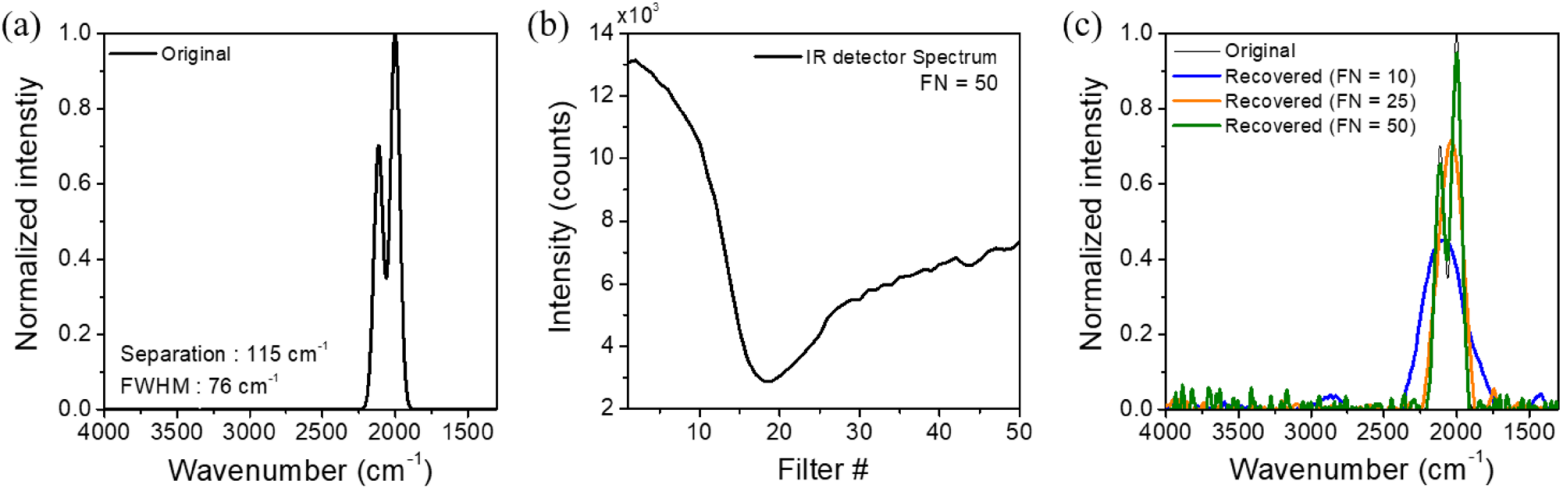
(**a**) Target spectrum consisting of a doublet of two Gaussian peak curves with an FWHM of 76 cm^−1^ and separated by 115 cm^−1^ from each other, (**b**) intensity profile measured from 1D IR detector array when the number of filters (FN) = 50, and (**c**) recovered spectra with different FN using Tikhonov regularization.

In order to evaluate the feasibility of the fabricated filter array for on-chip spectrometer applications, the measured filter transmission spectra were substituted for D_i_(λ) of the Tikhonov regularization algorithm and the spectrum reconstruction was tested. Figure 7 shows an example of spectrum reconstruction performed using the measured filter function shown in Fig. 5b. The target spectrum was assumed to be a doublet consisting of two Gaussian peak curves with an FWHM of 76 cm^−1^ and separated by 115 cm^−1^ from each other. When the target spectrum reaches the photodetector array through the stopband-type filter array, the signal intensities generated from each pixel of photodetectors are determined by Eq. (1). Figure 7b displays the calculated intensity distribution, which shows a considerably broadened and distorted reverse curve from the target one. This is attributed to the fact that the fabricated filter has a stopband characteristic with a broad linewidth and the spectral overlap between neighboring filters occurs severely.

For the convenience of calculation, it is assumed here that the responsivity of the infrared photodetector is constant irrespective of the wavelength. The number of filters was changed from 50 to 25 and 10 to evaluate its effect on the spectrum reconstruction and the results are shown in Fig. 7c. When the spectra of all 50 filters are used as the filter function, it is observed that the target spectrum is almost completely reconstructed, clearly resolving the doublet peaks. However, as the number of filters decreases, that is, when only 1/2 and 1/5 of the total filters are used, it can be seen that the doublet peaks cannot be resolved anymore and appear as a single overlapped peak curve that is increasingly broadened as the number of filters decreases. Consequently, increasing the number of filters constituting the filter array is advantageous for enhancing the resolution of spectrum reconstruction. On the other hand, although not shown here, if the object has a broad spectrum, the spectral reconstruction can be sufficiently well achieved even with a smaller number of filters.

**Figure 8:**
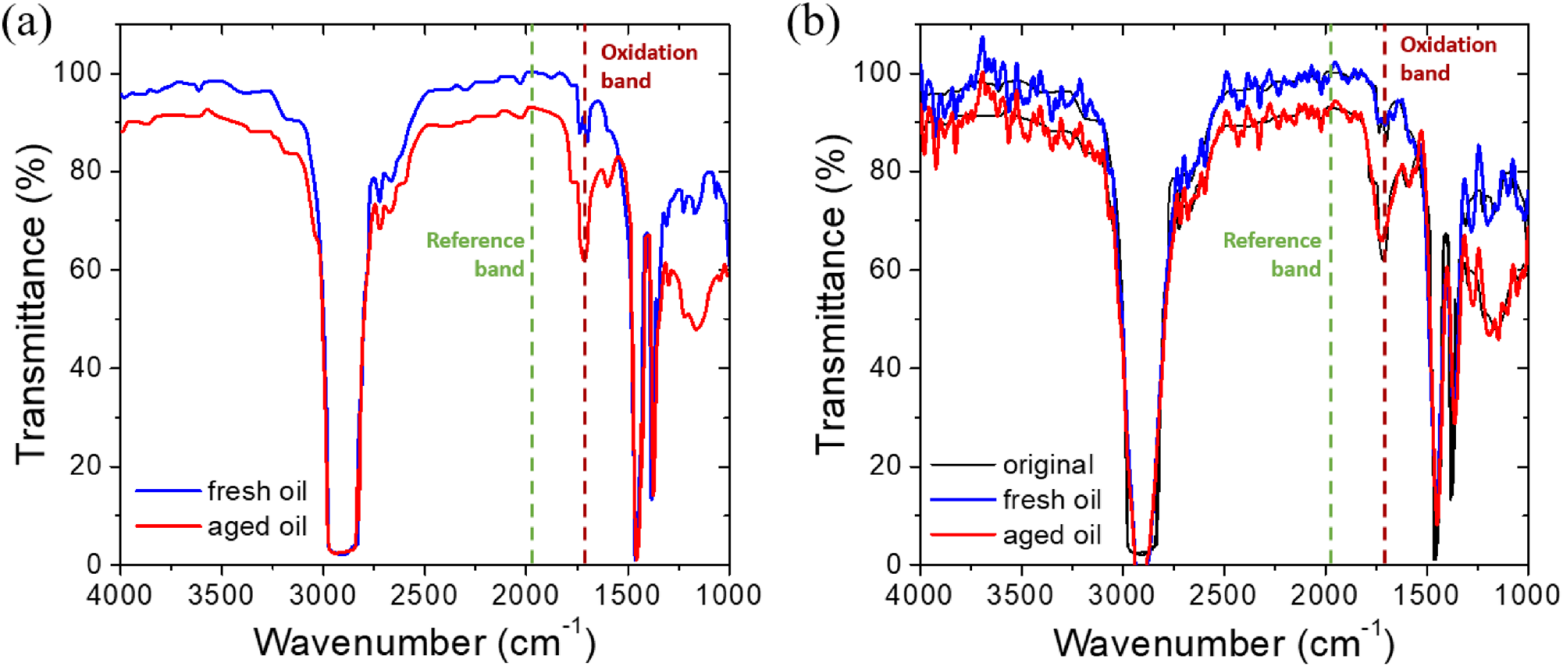
(**a**) FTIR spectra of fresh and aged oils (reproduced with permission from Ref.^38^ where the aged oil was obtained by artificially deteriorating an engine oil SAE 10W40 according to a standard CEC method^40^ at 160 °C for 8 days). (**b**) The recovered spectra using Tikhonov regularization, where thin black solid lines indicate the original spectra.

As a more practical example, the application to the IR spectroscopic sensor that monitors the degree of oil aging was evaluated and the results are shown in Fig. 8. As a standard method for oil condition monitoring, a method of comparing the difference in FTIR spectrum between fresh and aged oils has been used. Figure 8a shows the typical FTIR transmission spectra observed in a fresh and an aged oil. As the oil oxidizes, a characteristic infrared absorption peak (transmittance dip) is generated around 1710 cm^−1^ due to the formation of carboxylic acids and other compounds containing carbonyl group (C=O), and the magnitude of absorption increases according to the degree of oxidation^38^. A baseline at 1970 cm^−1^, which is not affected by oil aging, can be used as a reference to obtain the relative absorbance and quantitatively determine the degree of oxidation from it.

Figure 8b shows the result of performing the spectrum reconstruction assuming that the FTIR spectra of fresh and aged oils in Fig. 8a are used as the target object spectra and pass through the filter array fabricated in this study. Although the reconstructed spectra are somewhat noisy, it is noted that the overall characteristic spectral features of the target object are successfully recovered. In particular, the increase in absorption in the oxidation band due to oil oxidation can be distinguished without much difficulty. This confirms that it is possible to monitor the IR spectral change due to the oil aging with the stopband filter array based on the plasmonic nanodisc arrays fabricated in this study. The overall reconstruction quality was also quantified using a relative reconstruction error *ε* defined as follows^37,39^:

4 $$ \varepsilon = \frac{{\left\| {s_{\alpha } - s} \right\|{}_{2}}}{{\left\| s \right\|_{2} }} \times 100 $$

This equation represents how much the reconstructed spectrum deviates from the original target spectrum as a relative ratio of *l*_2_ norms. The evaluated errors are 5.33% and 5.40% for the fresh and aged oils, respectively. It should be noted that the noise reduction and the reconstruction resolution can be greatly enhanced by simply increasing the number of constituent filters. From this, it is verified that the mid-IR multispectral filter array provided in this study, when combined with the signal reconstruction technique, is valid for use in a miniaturized on-chip spectrometer which has great potential for on-site analysis applications.

## Conclusions

In this study, we employed the MNDA filters with a stopband characteristic in the transmissive mode operation of an on-chip spectrometer and presented their potential for the on-chip spectrometer applications. The effects of the geometrical configuration of the metal nanodisc array and the optical properties of constituent materials on the spectral response on the filter were analyzed systematically in terms of the duty cycle and period of the nanodisc array. It was found that the metal nanodiscs array with a duty cycle of 50% exhibited a narrow linewidth and sufficient dip depth, and the refractory metals in the mid-infrared region, whose optical behavior follows the Drude free electron model, can also be used as alternative plasmonic materials. The multispectral filter array based on a periodic lattice of Al nanodiscs was successfully fabricated by using soft-mold reverse-nanoimprint lithography and a subsequent lift-off process. The transmission spectra of individual filters constituting the filter array were measured in the wavenumber range of 4000–1000 cm^−1^ (2.5–10 μm) using an FTIR microscope. With the measured filter function, we tested the feasibility of target spectrum reconstruction through digital signal processing (DSP) based on the Tikhonov regularization method for an ill-posed linear problem and examined the effect of the number of filters on the spectral reconstruction resolution. As a more practical example, the application to the IR spectroscopic sensor that monitors the degree of oil aging was evaluated as well. These results not only verify that the mid-IR multispectral filter array provided in this study, when combined with the signal reconstruction technique, is valid for use in a miniaturized on-chip spectrometer which has great potential for on-site analysis applications but also provide effective guidance for the filter design.

## Methods

### Fabrication of Al nanodiscs array

In this study, the multispectral filter array based on a periodic lattice of Al nanodiscs was fabricated by using a soft-mold reverse-nanoimprint lithography and subsequent lift-off process. Figure 3 illustrates the schematic diagram of the overall procedure. The Si master stamps were prepared to have a series of multiple arrays of a triangular lattice of circular trenches of 200 nm depth with different periods. The filter arrays were designed in both 1D and 2D formats. The period varies from 1 to 3 μm at intervals of 40 nm and the duty cycle was fixed at 50%.

The Si master stamp was pre-coated with a hydrophobic self-assembled monolayer (SAM) of 1H, 1H, 2H, 2H-perfluorodecyltrichlorosilane (FDTS) diluted in an n-Hexane solvent so that the poly-dimethylsiloxane (PDMS) replica mold can be easily released from it. The PDMS prepolymer (Sylgard 184, Dow Corning) was mixed with curing agent in a mass ratio of 10:1, poured onto the relief surface of the master stamp, and degassed for about 30 min in a vacuum desiccator to remove air bubbles. Then the PDMS mold was baked in a dry oven for 1 h at the temperature of 95 °C and carefully peeled off from the stamp.

To facilitate the subsequent lift-off process, the undercut forming layer (LOR 3B, Microchem) was spin-coated at 3000 rpm for 30 s on a double-side polished Si substrate. Several drops of imprint resin of hydrogen silsesquioxane (HSQ, FOX16, Dow Corning) diluted 11 wt% in Methyl isobutyl ketone (MIBK, Sigma Aldrich) solvent were cast on the PDMS mold and spin-coated at 3000 rpm for 30 s. Then the LOR 3B pre-coated Si substrate was placed face-down on it and pressed at a pressure of 2 bar for 5 min to transfer the patterned HSQ resin onto the substrate. After detaching the PDMS mold, a two-step reactive ion etching (RIE) process was carried out to remove the HSQ residue and to etch the LOR 3B located below it. For the removal of HSQ residue, a mixed gas of CHF_3_ and CF_4_ was used, and O_2_ gas was used to etch the LOR 3B layer to yield an undercut pore opening through the substrate. Then, the Al thin film of 50 nm thickness was e-beam evaporated on the patterned resin. Finally, the lift-off process was carried out by immersing the sample in dimethylformamide (DMF), a weak base of pH 8 for more than 1 h to create the Al nanodisc array.

### FT-IR transmission measurement

The transmittance spectra of the fabricated filter array were measured in the wavenumber range of 4000–1000 cm^−1^ (2.5–10 μm) using an FTIR microscope (Spotlight 150, PerkinElmer). The sampling area was adjusted to 200 μm × 200 μm using a variable aperture and the relative transmittance spectra for each filter were obtained by normalizing the spectra with the one of bare Si substrate.
